# Clinical Outcomes of Transcatheter Aortic Valve Replacement (TAVR) vs Surgical Aortic Valve Replacement (SAVR) in Patients With Sarcoidosis

**DOI:** 10.7759/cureus.62477

**Published:** 2024-06-16

**Authors:** Muhammad Z Khan, Rene Alvarez, Mohammad Alfrad Nobel Bhuiyan, Abu Saleh Mosa Faisal, Parker O'Neill, Muhammad Siddiqui, Praneet Kaki, Sona Franklin, Muhammad Waqas, Hadia Shah, Eyad I Kanawati, Mohammed Murtaza

**Affiliations:** 1 Cardiology, Thomas Jefferson University, Philadelphia, USA; 2 Heart Failure, Thomas Jefferson University Hospital, Philadelphia, USA; 3 Biostatistics, Louisiana State University (LSU) Health Shreveport, Shreveport, USA; 4 Biostatistics, Louisiana State University Health Sciences Center, Shreveport, USA; 5 Internal Medicine, Thomas Jefferson University, Philadelphia, USA; 6 Medicine, Sidney Kimmel Medical College, Thomas Jefferson University, Philadelphia, USA; 7 Internal Medicine, Jefferson Abington Hospital, Abington, USA; 8 Internal Medicine, Swat Medical College, Swat, PAK; 9 Medicine and Surgery, Saidu Medical College Swat, Swat, PAK

**Keywords:** aortic valve surgery, extra pulmonary manifestations of sarcoidosis, systemic sarcoidosis, as: aortic stenosis, tavr( transcatheter aortic valve replacement), surgical aortic valve replacement (savr), complications of sarcoidosis

## Abstract

Introduction

Data regarding clinical outcomes after transcatheter aortic valve replacement (TAVR) vs surgical aortic valve replacement (SAVR) in patients with sarcoidosis is lacking. This study aims to clarify the clinical outcomes of TAVR vs SAVR in patients with sarcoidosis.

Methods

Data was collected from the National Inpatient Sample database from 2016-2019 using validated ICD-10-CM codes for sarcoidosis, TAVR, and SAVR. Patients were divided into two cohorts: those who underwent TAVR and those who underwent SAVR. Statistical analysis was performed using Pearson's chi-squared test to determine clinical outcomes of TAVR vs SAVR in patients with sarcoidosis.

Results

The prevalence of sarcoidosis was 0.23% among total study patients (n=142,420,378). After exclusions, the prevalence of TAVR was 650 (49%) and SAVR was 675 (51%) in patients with sarcoidosis. Patients who underwent TAVR were on average older (74 vs 65 years old, p=0.001), and more likely to be female (57 vs 40%, p<0.001) compared to patients who underwent SAVR. The TAVR cohort had higher rates of congestive heart failure (CHF) (77.7 vs 42.2%, p=0.001), chronic kidney disease (CKD) (42.3 vs 24.4% p=0.001), anemia (5.4 vs 2.2%, p=0.004), percutaneous coronary intervention (PCI) (1.5 vs 0%, p=0.004), and hypothyroidism (31.5 vs 16.3%, p=0.001) compared to the SAVR cohort. Inpatient mortality post-procedure was higher in the SAVR cohort compared to the TAVR cohort (15 vs 0, p=0.001). Regarding post-procedure complications, respiratory complications were more common in the SAVR cohort (4.4 vs 0%, p=0.001), while TAVR was associated with a higher incidence of permanent pacemaker (PPM) insertion (2.15 vs 0.8%, p=0.001). There was no statistical difference in the development of acute kidney injury (AKI) (0.8 vs 1.5%, p=0.33), AKI requiring hemodialysis (0 vs. 0.7%, p=0.08), or stroke (0.8 vs 0.7, p=1) post-procedure between the two cohorts.

Conclusion

This study found that in the sarcoidosis population, TAVR was associated with reduced mortality, shorter hospital length of stay, and lower hospitalization costs in comparison to SAVR.

## Introduction

Sarcoidosis is a multisystem granulomatous disorder that typically presents in patients between the ages of 20 and 60. Sarcoidosis most frequently involves the lungs, but up to 30 percent of patients present with extrathoracic manifestations of sarcoidosis [1]. In systemic sarcoidosis, patients present with heart complications such as advanced conduction disease, heart failure, and valvular disease [1]. A clinicopathologic study has found the incidence of valvular involvement in patients with systemic sarcoidosis to be 5% [1,2]. Some of these patients may require aortic valve replacement using either transcatheter aortic valve replacement (TAVR) or surgical aortic valve replacement (SAVR). Patients with cardiac sarcoidosis have a higher incidence of conduction system defects and valvular disease compared to the general population, with higher rates of implantable cardioverter-defibrillator (ICD) and permanent pacemaker (PPM). This is possibly due to the presence of noncaseating granulomas in the myocardium that may disrupt natural electrical conduction. Due to the complications arising from myocardial granuloma formation, patients with sarcoidosis and concomitant aortic stenosis (AS) may experience higher rates of complication and greater hospitalization costs from aortic valve replacement. This study aims to determine the clinical and economic outcomes of TAVR vs SAVR in patients with sarcoidosis. Our primary objective is to compare inpatient and outpatient mortality rates between TAVR and SAVR patients with sarcoidosis. Our secondary objective is to compare the length of stay, hospitalization costs, as well as post-procedure complications in sarcoidosis patients who underwent TAVR and SAVR.

## Materials and methods

Data source

This study utilized data from the National Inpatient Sample (NIS) database, which is one of the largest inpatient database systems in the United States. The NIS database collects data from 48 states and is representative of more than 97% of the United States population, accounting for an average of 7-8 million discharges and more than 35 million hospitalizations annually. Patient demographics, comorbidities, complications, and primary and secondary discharge diagnoses can be obtained for each patient admission. The International Classification of Disease, 10th revision, Clinical Modification (ICD 10-CM) codes were used to identify the NIS database's diagnosis [3]. NIS data includes the charge-to-cost ratio, where charges represent the hospital bill at the end of the admission, and cost represents the cost of service, including utility, supplies, and wages. We included patients older than 18 years with a primary discharge diagnosis of sarcoidosis between 2016 and 2019. Study subjects were obtained from the National Inpatient Sample Database between the years 2016 and 2019. Only patients over the age of 18 with sarcoidosis were included in the study. Subjects excluded were patients under the age of 18 years, those who did not undergo TAVR or SAVR, and those who underwent both TAVR and SAVR. ICD 10-CM codes were utilized to extract patients with sarcoidosis from the NIS database. From within this subset of patients, ICD codes were again utilized to delineate those who underwent TAVR and SAVR, as well as to identify those with specific comorbidities and baseline characteristics. The study cohort was derived from a de-identified and publicly available database; hence, the study was considered exempt from formal Institutional Review Board approval.

Diagnostic codes for sarcoidosis and other comorbidities

Clinical Classifications Software (CCS) codes for sarcoidosis were used to extract data from the NIS database which provides up to 30 CCS diagnoses for each inpatient visit (See Supplementary Table in the Appendix). We extracted data on patients with sarcoidosis who underwent either TAVR or SAVR using appropriate ICD 10-CM diagnostic codes in primary and secondary diagnosis (See Supplementary Table in the Appendix). Furthermore, we documented the following comorbidities: hypertension, diabetes, congestive heart failure (CHF), anemia, hypothyroidism, coronary artery disease (CAD), smoking, liver disease, and chronic kidney disease (CKD). The clinical outcomes measured were in-hospital mortality and complications post-procedure. The economic outcomes included the length and cost of TAVR/SAVR hospitalization.

Statistical analysis

The data analysis and extraction were done using SAS statistical software version 9.4 (SAS Institute Inc., Cary, NC, USA). All continuous variables were compared using the Student's T-test. These variables were presented as a mean ± standard deviation (SD) for normally distributed variables. Median and interquartile ranges were used for non-Gaussian distributed variables. Categorical variables were analyzed using the Pearson’s chi-square test. These variables were presented as a weighted frequency in percentages. A p-value of <0.05 was considered statistically significant.

## Results

**Figure 1:**
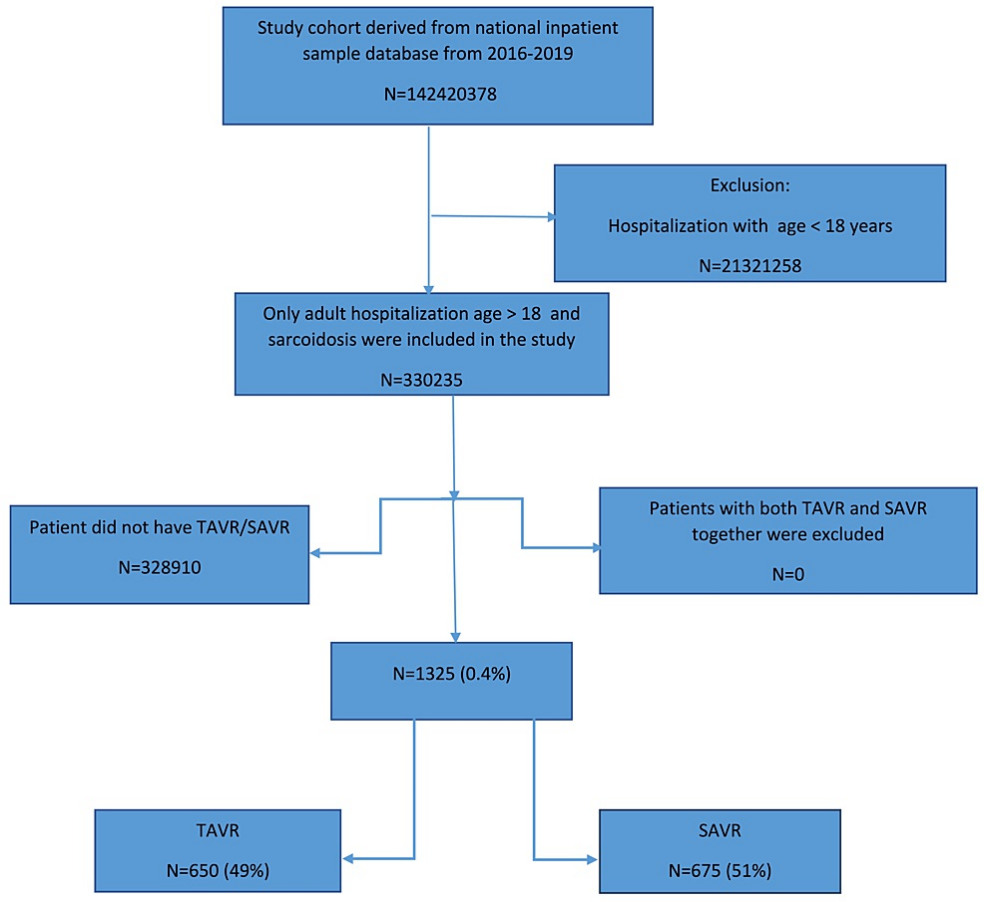
Flow chart of the study selection process TAVR=transcatheter aortic valve replacement; SAVR=surgical aortic valve replacement

The NIS database included 142,420,378 patients between 2016 and 2019, among which 327,567 patients (0.23%) had a diagnosis of sarcoidosis. Our study included 1,325 patients with sarcoidosis who underwent aortic valve replacement, of which 650 patients (49%) underwent TAVR, and 675 patients (51%) underwent SAVR (Figure 1).

Baseline characteristics

A comparison of baseline characteristics between the TAVR and SAVR cohorts is shown in Table 1. Patients who underwent TAVR were on average older (74 vs 65 years old, p=0.001) and more likely to be female (57 vs 40%, p<0.001) compared to patients who underwent SAVR. The TAVR cohort had higher rates of CHF (77.7 vs 42.2%, p=0.001), CKD (42.3 vs 24.4%, p=0.001), anemia (5.4 vs 2.2%, p=0.04), percutaneous coronary intervention (PCI) (1.5 vs 0%, p=0.04), and hypothyroidism (31.5 vs 16.3%, p=0.001) compared to the SAVR cohort. On the other hand, the prevalence of coronary artery bypass grafting (CABG) (24.4 vs 0%, p=0.001) and chronic obstructive pulmonary disease (COPD) (2.2 vs 0.8%, p=0.05) was higher in the SAVR cohort than in the TAVR cohort.

**Table 1:** Patient-level characteristics of TAVR vs SAVR in cardiac sarcoidosis. A p-value of <0.05 was considered statistically significant. AF=atrial fibrillation; CABG=coronary artery bypass surgery; CAD=coronary artery disease; CHF=congestive heart failure; CKD=chronic kidney disease; COPD=chronic obstructive pulmonary disease; MI=myocardial infarction; PCI=percutaneous coronary intervention; PVD=peripheral vascular disease; SAVR=surgical aortic valve replacement; TAVR=transcatheter aortic valve replacement.

Characteristics	TAVR N=650	SAVR N=675	P-Value
Age	74.06 (MD = 75)	65.26 (MD = 66)	<0.001
Gender			<0.001
Male (%)	275 (42.3%)	400 (59.3%)	-
Females (%)	375 (57.7%)	275 (40.7%)	-
Race			<0.001
Whites (%)	570 (87.7%)	465 (68.9%)	-
Blacks (%)	55 (8.5%)	130 (19.3%)	-
Others (%)	10 (3.8%)	45 (11.8%)	-
Liver Disease (%)	50 (7.7%)	35 (5.2%)	0.08
Obesity (%)	55 (8.5%)	60 (8.9%)	0.8
CKD (%)	275 (42.3%)	165 (24.4%)	<0.001
COPD (%)	5 (0.8%)	15 (2.2%)	0.05
CAD (%)	30 (4.6%)	25 (3.7%)	0.5
PCI (%)	10 (1.5%)	0 (0%)	0.004
CABG (%)	0 (0%)	165 (24.4%)	<0.001
Hypertension (%)	1101 (6.9%)	285 (42.2%)	<0.001
PVD (%)	20 (3.1%)	25 (3.7%)	0.6
Hypothyroidism (%)	205 (31.5%)	110 (16.3%)	<0.001
CHF (%)	505 (77.7%)	285 (42.2%)	<0.001
Electrolyte derangement (%)	10 (1.5%)	45 (6.7%)	<0.001
Smoking (%)	140 (21.5%)	140 (20.7%)	0.8
Anemia (%)	35 (5.4%)	15 (2.2%)	0.004
AF (%)	240 (36.9%)	340 (50.4%)	<0.001
Dyslipidemia (%)	375 (57.7%)	445 (65.9%)	0.002
Old MI (%)	65 (10%)	40 (5.9%)	0.008

Clinical outcomes

Mortality

Inpatient mortality post-procedure was higher in the SAVR cohort compared to the TAVR cohort (15 vs 0, p=0.001, Table 2). Annual trends in mortality demonstrated that in the years 2011 and 2014, mortality rates were significantly higher in the SAVR group. In addition, a comparison of the TAVR group to the SAVR group revealed that the mean length of stay (4.4 and 10.7 days, p=<0.000001) and mean cost ($199,720 and $237,872, p=0.03) were lower in the TAVR cohort.

**Table 2:** Clinical outcomes of TAVR vs SAVR in cardiac sarcoidosis A p-value of <0.05 was considered statistically significant. LOS=length of stay; SAVR=surgical aortic valve replacement; TAVR=transcatheter aortic valve replacement; USD=US Dollars.

-	Number of Deaths	P-value	Mean LOS (days) [95% CI]	Median LOS	P-value	Mean Cost (USD) (95% CI)	Median Cost (USD)	P-value
TAVR	0	<0.001	4.4 (2-3)	2	<0.001	199720 (154242-191043)	172103	0.03
SAVR	15		10.7 (8-10)	8		237872 (172478-212374)	190963	

Complications

Notably, a higher rate of PPM insertions was associated with the TAVR cohort (14 vs 6, p=0.001, Table 3), whereas respiratory complications were only found among the SAVR cohort (0 vs 30, p=<0.0001). There was no significant difference in the development of acute kidney injury (AKI), AKI requiring dialysis, or cerebral infarction between groups. In addition, no patients in either group developed thromboembolic events, cardiac complications, infections, or vascular complications.

**Table 3:** Complications of TAVR vs SAVR in cardiac sarcoidosis A p-value of <0.05 was considered statistically significant. AKI=acute kidney injury; SAVR=surgical aortic valve replacement; TAVR=transcatheter aortic valve replacement.

Variable	TAVR	SAVR	P-Value
	650	675	
Permanent Pacemaker	14 (2.15%)	6 (0.8%)	0.001
AKI	5 (0.8%)	10 (1.5%)	0.33
AKI requiring hemodialysis	0	5 (0.7%)	0.08
Respiratory	0	30 (4.4%)	<0.001
Cerebral infarction	5 (0.8%)	5 (0.7%)	1

Economic outcomes

Cost of Hospitalization and Length of Stay

**Figure 2:**
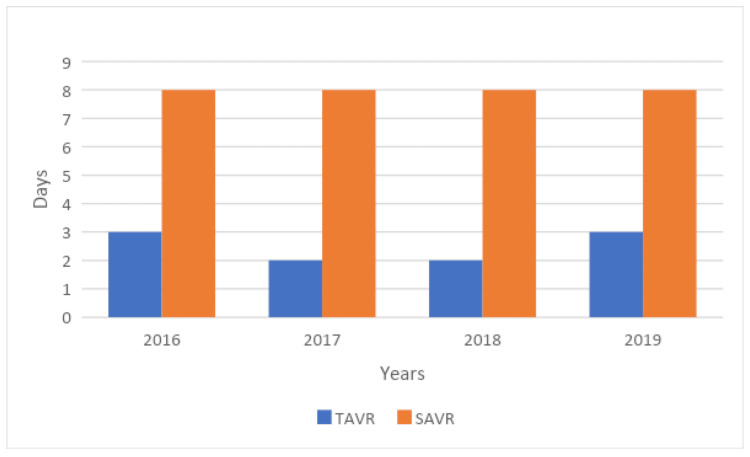
Trends in length of stay for TAVR vs SAVR in sarcoidosis patients SAVR=surgical aortic valve replacement; TAVR=transcatheter aortic valve replacement.

**Figure 3:**
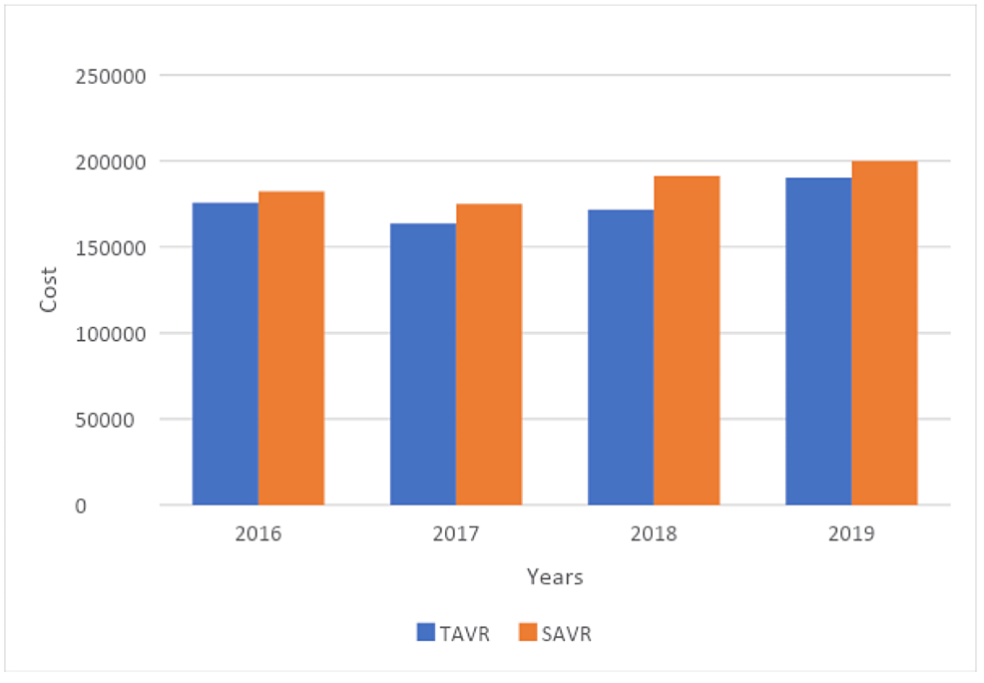
Trends in cost for TAVR vs SAVR in sarcoidosis patients SAVR=surgical aortic valve replacement; TAVR=transcatheter aortic valve replacement.

Hospitalization costs associated with SAVR were higher than those associated with TAVR ($190,963 vs. $172,103, p=0.001). The length of stay was shorter for patients undergoing TAVR (2 vs. 8 days, p=0.001). The annual trends of mean length of stay and hospitalization cost were compared in Table 4, Table 5, Figure 2, and Figure 3, respectively. These figures showed that the annual trends of mean length of stay and hospitalization cost were high in the SAVR group as compared to TAVR.

**Table 4:** Yearly cost among TAVR vs SAVR p-value of <0.05 was considered statistically significant. SAVR=surgical aortic valve replacement; TAVR=transcatheter aortic valve replacement.

Year	TAVR	SAVR	
2016	205830.5±35698.3	248161.8±78934.2	<0.001
2017	180396.6±48298.5	226956.1±56987.4	<0.001
2018	192637.6±58976.4	228768.2±87632.3	<0.001
2019	223975.3±38975.4	251297.2±45678.6	<0.001

**Table 5:** Length of stay yearly among TAVR vs SAVR. p-value of <0.05 was considered statistically significant. SAVR=surgical aortic valve replacement; TAVR=transcatheter aortic valve replacement.

Year	TAVR	SAVR	P-value
2016	5.6±2.4	10.4±3.4	<0.001
2017	4.2±1.5	11.2±4.7	<0.001
2018	3.3±1.4	10.1±4.2	<0.001
2019	5.2±2.1	11±4.8	<0.001

## Discussion

This study aimed to determine the clinical and economic outcomes of TAVR vs SAVR in patients with sarcoidosis and AS requiring intervention. Our results demonstrated that TAVR is associated with reduced mortality, shorter length of hospital stay, and lower hospitalization costs compared to SAVR. Furthermore, the SAVR cohort experienced a greater incidence of respiratory complications than the TAVR cohort, while undergoing TAVR was associated with more post-procedure PPM insertions. There was no significant difference in the incidence of stroke, AKI, or dialysis initiation between the two cohorts.

A brief overview of sarcoidosis and AS

Sarcoidosis is an infiltrative disease mediated by T helper 1 cells in response to a currently unknown antigen that leads to a cytokine storm with the formation of non-caseating granulomas [3,4]. Cardiac sarcoidosis (CS) is a relatively rare manifestation of sarcoidosis, with aortic valve involvement occurring even more infrequently. Silverman et al. found the incidence of valvular involvement in patients with systemic sarcoidosis to be around 5% [1]. A clinicopathologic study by Roberts et al. found that the left ventricle was the most common site for granuloma formation, and ventricular arrhythmias were the most frequent complication of granuloma formation [5]. The incidence of aortic valve involvement in CS is rare with only a few case reports currently present in the literature. Netsu et al. described a 75-year-old male with sarcoidosis who presented with severe AS and successfully underwent balloon aortic valvuloplasty as a bridge to SAVR [6]. Koizumi et al. highlighted the case of a 78-year-old with CS who required two aortic valve replacements due to recurrent structural valve deterioration [7]. Due to the paucity of evidence surrounding the treatment of AS in sarcoid patients, our study aimed to determine the clinical and economic outcomes of TAVR vs SAVR in patients with sarcoidosis and AS requiring intervention.

The 2020 Joint American College of Cardiology and American Heart Association Guidelines recommend SAVR for symptomatic and asymptomatic patients with severe AS who are either younger than 65 years of age or who have a life expectancy greater than 20 years [8-11]. TAVR is recommended for symptomatic patients over 80 years of age with severe AS, or younger patients with a life expectancy of less than 10 years [12]. Recent clinical trials have suggested that TAVR may yield similar outcomes to SAVR in intermediate and low-risk surgical candidates [12]. As the indications for TAVR and SAVR overlap, it is imperative to distinguish the outcomes between the two procedures in patients with sarcoidosis.

Clinical outcomes (mortality and complications)

Our results showed that patients with sarcoidosis experienced reduced mortality when undergoing TAVR compared to SAVR. Consistent with our findings, a large randomized controlled trial of 1,000 low-surgical-risk patients with severe AS conducted by Mack et al. found that TAVR resulted in lower rates of death, stroke, new-onset atrial fibrillation, and shorter postoperative hospitalization than SAVR [11]. Lauck et al. showed that the magnitude of the 30-day mortality benefit associated with TAVR increased from 2012 to 2016; however, mortality rates continued to decline from 2017 to 2019 among those undergoing TAVR. The study also showed that TAVR was associated with a lower mortality rate than SAVR throughout all years [12].

Literature shows that patients who underwent SAVR experienced a higher rate of respiratory complications. After comparing our study with the literature, our results indicate that sarcoidosis patients experienced the same complication when patients go for TAVR and SAVR procedures. There was no significant difference in stroke, AKI, or dialysis initiation between the cohorts. Our results are consistent with previous studies, including a large meta-analysis involving 12,467 patients which found TAVR to be associated with a higher incidence of PPM insertions than SAVR [13], and no significant difference in the two-year incidence of stroke between patients undergoing TAVR or SAVR. The incidence of pacemaker in the TAVR group is high because of the development of atrioventricular conduction abnormalities. After comparing our study with the literature, our results indicate that sarcoidosis does not significantly influence the rate of complications between TAVR and SAVR procedures.

Economic outcomes (cost of hospitalization and length of stay)

This study found that patients who underwent TAVR had a significantly shorter hospital stay (average of two days) compared to those who underwent SAVR (average of eight days). Our results were consistent with previous studies demonstrating that TAVR is associated with a shorter hospitalization period. For instance, a comparative study by Arora et al. involving 18,099 patients found that patients undergoing TAVR had an average stay of 4.6 days compared to 6.8 days for the SAVR cohort [14]. Moreover, TAVR was associated with more frequent home discharges (instead of to a skilled nursing facility) than patients who underwent SAVR. Likely causes for increased length of hospitalization in the SAVR cohort include the need for longer physical recovery from sternotomy and chest tube placement involved in surgical repair, and the use of general anesthesia (as opposed to the conscious sedation used for TAVR), leading to extended post-operative intensive care unit stays [15,16]. Our results indicate that concomitant sarcoidosis does not drastically impact the length of stay for patients undergoing aortic valve replacement and that TAVR continues to provide a shorter hospitalization than SAVR.

Our study found that, on average, TAVR was associated with significantly lower hospitalization costs compared to SAVR. Consistent with previous research, Baron et al. performed a retrospective cohort study capturing data from the Medicare database that included 9,746 patients who underwent aortic valve replacement and found that the average TAVR hospitalization cost was $65,594 compared to the SAVR hospitalization cost of $91,005 [17]. Furthermore, the one-year follow-up costs were also lower in the TAVR cohort than in the SAVR group. A cost analysis of the PARTNER trial found that 25% of TAVR hospitalization costs were due to procedural complications including major bleeding, arrhythmias, and renal failure [18]. With the increased frequency of TAVR procedures leading to improved operator proficiency, there has been a steady decline in hospitalization costs associated with TAVR [18]. Another reason for TAVR to be less expensive than SAVR is that TAVRs are frequently performed in catheterization laboratories instead of operating rooms, which are associated with lower operating costs [19]. Our study highlighted that patients with sarcoidosis experience similar hospitalization costs to previously studied patient populations, with TAVR being associated with reduced costs compared to SAVR.

Limitations

Our study has several limitations. First, our study only evaluated patients in the US, thus our findings may not be able to be extrapolated to international populations, especially when discussing hospitalization costs and length of stay. Thus, our findings may not be generalizable to many international populations. Further studies would be needed internationally to truly clarify if our findings were comparable to other populations. As the NIS database is limited to the inpatient population, adequately determining long-term outcomes of patients proves difficult. Furthermore, as ICD 10-CM codes were used to identify study patients, coding inaccuracies are certainly possible, potentially leading to possible biases in our results. Lastly, the inherent biases associated with retrospective studies are understood. In order to reduce these biases, a prospective study based on our findings would need to be conducted. Finally, our data also relied upon physicians accurately inputting correct diagnosis codes for patients, which could have potentially led to exposure and outcome misclassification.

The strength of our study is the utilization of a large, comprehensive, and accessible national database. In addition, our study evaluated a sizeable population with multiple comorbidities and baseline characteristics which would be generalizable to the US population.

## Conclusions

This study found that TAVR was associated with reduced mortality, shorter length of hospital stay, and lower hospitalization costs compared to SAVR in the sarcoidosis population. Our outcomes in the sarcoidosis population are consistent with previously studied general patient populations, indicating that the presence of sarcoidosis does not significantly affect outcomes after TAVR vs SAVR. Therefore, choosing the optimal procedure should be based on other prognostic factors. As our study shows reduced mortality and length of stay in TAVR procedure when compared to SAVR, clinicians might consider TAVR as a potentially more beneficial therapeutic modality. In addition, TAVR may be preferable considering the lower overall cost. TAVR procedure itself may necessitate PPM placement, thus the addition of a Holter monitor or loop recorder may be needed to identify these heart block patients earlier.

## Appendices

**Table 6:** Supplementary Table AKI=acute kidney injury; CABG=coronary artery bypass grafting; IABP=intra-aortic balloon pump; NSTEMI=non-ST-elevation myocardial infarction; PCI=percutaneous coronary intervention; SAVR=surgical aortic valvular replacement; STEMI=ST-elevation myocardial infarction; TAVR=transcatheter aortic valve replacement

COMORBIDITIES	ICD 10-CM CODES
Dyslipidemia	E78, E78.5, E78.4,
Chronic Obstructive Pulmonary Disease	J441, J449, J44, J440,
Carotid Artery Disease (occlusion and stenosis)	I65.2, I65.22, I65.29, I65.21
Hypertension	I10
Old Myocardial Infarction	I25.2
Peripheral Vascular Disease	I739, I7389, I73
Hypothyroidism	E03, E031, E032, E033, E038, E039
Diabetes Mellitus Type 1&2	E11.0, E11.00, E11.3, E11.319, E11.42, E11.610, E11.621, E11.63, E11.9 E10.36, e10.41, E10.21, E10.35,e10.610
Obesity	E660, E6601, E6609, E661, E662, E668, E669
Chronic Kidney Disease	N18, n18.6, N18.1, N18.9, N18.2, n18.3, N18.4, N18.5,
Liver Disease	K700, K7031, K702, K703, K704, K705, K706, K707, K70, k701, k7011, K74, K745, K743, K7460
Sarcoidosis	D86, D86.2, D86.87, D86.3, D86.89, D86.1
Electrolyte Derangement	E870, E871, E872, E873, E874, E875, E876, E87
Smoking	Z87891, F17, F17210, F17203, F17200, F1720, F172
Anemia	D50, D51, D52, D53, D55, D56, D57, D58, D50, D60, D61, D62, D63, D64
Coronary Artery Disease (STEMI AND NSTEMI)	I200, I214, I21, I12109, I121.02, I2111,I2111, I211, I21, I210
Amyloidosis	E854, E850, E851, E852, E853, E854, E8581, E8582, E8589, E859,
Atrial Fibrillation	I48, I48.1, I48.2, I48.91, I48.92, I48.9
Congestive Heart Failure	I50.1, I50.20, I50.21, I50.22, I50.23, I50.30, I50.31, I50.32, I50.33, I50,40, I50.41, I50.42, I50.43
PROCEDURES	ICD 10-CM CODES
PCI	0270, 0271, 0272, 0273
CABG	0213093,02130A8, 02130AF, 02130K3, 0213498, 02134AC, 02134AF, 02134AF, 02130A9, 02130AC 0210099, 02100A3, 02100J9, 02100JC, 02100K3, 02100KF, 02100ZF 0211099, 02110AF, 02110J8, 02110JW, 02114A8, 02114J3, 02110A3.02110A8 0212099, 021209C, 02120AC, 02120AW, 02120J8, 02120K3, 20120Z8, 02120ZF
Impella Device	5A0221D, 5A0211D
IABP	5A02210, 5A02110
Sarcoidosis	D86, D86.2, D86.87, D86.3, D86.89, D86.1
SAVR	02RF0 (02RF07Z, 02RF08Z , 02RF0JZ, 02RF0KZ)
TAVR	02RF3 (02RF37H, 02RF37H, 02RF38H, 02RF38Z, 02RF3JZ, 02RF3JH, 02RF3KH, 02RF3KZ, ), 02RF4 (02RF47Z, 02RF48Z, 02RF4JZ, 02RF4KZ)
COMPLICATION	ICD 10-CM Codes
Vascular	T81718, T81718A, T81719, T91719A, T8172, T8172XA, T82837, T82837A
Cardiac	I9711, I97110, I9712, I97120, I9713, I97130, I9719, I9789
Respiratory	J951, J953, J95821, J95822, J9589
Infection	T8112, T8112XA, T814, T814XXA, T826, T826XXA, T827, T827XXA, T81.4XXS
Acute Kidney Injury	N990
Shock	R570, R578, T811, T8119XA, R571
Thromboembolic Event	T8281, T82817, T82817A, T82867, T82867A
Permanent Pacemaker Implantation	02H40JZ, 02H40NZ, 02H34JZ, 02H44NZ, 02H60NZ, 02H63JZ, 02H64JZ, 02H64NZ, 02H70JZ, 02H70NZ, 02H73JZ, 02H73NZ, 02H74JZ, 02HK3JZ, 02HL3NZ, 02HL4JZ, 02HNOJZ, 02HN4JZ
Cerebral Infarction (stroke)	I97820, I9782, I9781
AKI Requiring dialysis	5A1D00Z, 5A1D60Z, 3E1M39Z

## References

[1] Silverman KJ, Hutchins GM, Bulkley BH (1978). Cardiac sarcoid: a clinicopathologic study of 84 unselected patients with systemic sarcoidosis. Circulation.

[2] Birnie D, Ha A, Kron J (2018). Which patients with cardiac sarcoidosis should receive implantable cardioverter-defibrillators: some answers but many questions remain. Circ Arrhythm Electrophysiol.

[3] (2023). ICD-10. https://www.cms.gov/medicare/coding-billing/icd-10-codes.

[4] Thakker RA, Abdelmaseih R, Hasan SM (2021). Sarcoidosis and aortic stenosis: a role for transcatheter aortic valve replacement?. Curr Probl Cardiol.

[5] Roberts WC, McAllister Jr HA, Ferrans VJ (1977). Sarcoidosis of the heart. A clinicopathologic study of 35 necropsy patients (group I) and review of 78 previously described necropsy patients (group II). Am J Med.

[6] Netsu S, Omi K, Goto J, Sugawara S (2016). A case of severe aortic stenosis with sarcoidosis successfully treated with balloon aortic valvuloplasty to control heart failure. J Cardiac Fail.

[7] Koizumi S, Matsuura K, Kobayashi Y, Matsumiya G (2017). Low-gradient structural valve deterioration in a patient of cardiac sarcoidosis. J Cardiovasc Echogr.

[8] Otto CM, Nishimura RA, Bonow RO (2021). 2020 ACC/AHA guideline for the management of patients with valvular heart disease: executive summary: a report of the American College of Cardiology/American Heart Association Joint Committee on Clinical Practice Guidelines. Circulation.

[9] Leon MB, Smith CR, Mack M (2010). Transcatheter aortic-valve implantation for aortic stenosis in patients who cannot undergo surgery. N Engl J Med.

[10] Kapadia SR, Leon MB, Makkar RR (2015). 5-year outcomes of transcatheter aortic valve replacement compared with standard treatment for patients with inoperable aortic stenosis (PARTNER 1): a randomised controlled trial. Lancet.

[11] Mack MJ, Leon MB, Smith CR (2015). 5-year outcomes of transcatheter aortic valve replacement or surgical aortic valve replacement for high surgical risk patients with aortic stenosis (PARTNER 1): a randomised controlled trial. Lancet.

[12] Lauck SB, Baron SJ, Irish W (2021). Temporal changes in mortality after transcatheter and surgical aortic valve replacement: retrospective analysis of US Medicare patients (2012-2019). J Am Heart Assoc.

[13] Lou Y, Gao Y, Yu Y (2020). Efficacy and safety of transcatheter vs. surgical aortic valve replacement in low-to-intermediate-risk patients: a meta-analysis. Front Cardiovasc Med.

[14] Arora S, Strassle PD, Kolte D (2018). Length of stay and discharge disposition after transcatheter versus surgical aortic valve replacement in the United States. Circ Cardiovasc Interv.

[15] Dolansky MA, Xu F, Zullo M, Shishehbor M, Moore SM, Rimm AA (2010). Post-acute care services received by older adults following a cardiac event: a population-based analysis. J Cardiovasc Nurs.

[16] Toppen W, Johansen D, Sareh S (2017). Improved costs and outcomes with conscious sedation vs general anesthesia in TAVR patients: time to wake up?. PLoS One.

[17] Baron SJ, Ryan MP, Moore KA (2022). Contemporary costs associated with transcatheter versus surgical aortic valve replacement in Medicare beneficiaries. Circ Cardiovasc Interv.

[18] Arnold SV, Lei Y, Reynolds MR (2014). Costs of periprocedural complications in patients treated with transcatheter aortic valve replacement: results from the placement of aortic transcatheter valve trial. Circ Cardiovasc Interv.

[19] Carroll JD, Mack MJ, Vemulapalli S (2020). STS-ACC TVT registry of transcatheter aortic valve replacement. J Am Coll Cardiol.

